# Dietary sunflower hulls and protein levels affect performance, gut health, and litter quality in broiler chickens

**DOI:** 10.1016/j.vas.2026.100806

**Published:** 2026-08-10

**Authors:** N. Beigzadeh, S. Salari, F. Zaefarian, Sh. Hosseinifar

**Affiliations:** a Department of Animal Science, Animal Science and Food Technology Faculty, Agricultural Sciences and Natural Resources University of Khuzestan, Ahvaz, P.O. Box: 6341773637, Iran; b Adisseo France S.A.S. European Laboratory of Innovation Science & Expertise (ELISE), 20 rue Prosper Monnet, 69190, Saint Fons, France; c Department of Basic Sciences, Veterinary Medicine Faculty, Shahid Chamran University of Ahvaz, Ahvaz, Iran

**Keywords:** Broiler chickens, Insoluble fiber, Reduced crude protein, Nutrient digestibility, Gut morphology

## Abstract

This study evaluated the effects of different dietary levels of sunflower hulls (SFH) as an insoluble fiber source on growth performance, nutrient digestibility, intestinal morphology, cecal microbial populations, litter quality, and digesta transit time in broiler chickens fed varying levels of crude protein (CP). A total of 360 one-day-old broiler chicks were randomly assigned to a 3 × 2 factorial design comprising three SFH levels (0, 20, and 40 g/kg) and two CP levels (normal and 10% reduced). Broilers fed diets containing 40 g/kg SFH combined with normal CP exhibited the most favorable feed conversion ratio (FCR), whereas the poorest FCR was observed in those reared on the fiber-free, low-CP diets. Dietary inclusion of SFH progressively enhanced the apparent ileal digestibility (AID) of nitrogen, organic matter, and ether extract. Furthermore, improvements in villus height, width, and surface area in both the duodenum and jejunum were observed, as levels of SFH increased. These morphological advancements were accompanied by a significant shift in the cecal ecosystem, characterized by an increased population of beneficial *Lactobacillus* spp. Additionally, the inclusion of 40 g/kg SFH effectively reduced litter nitrogen content. In conclusion, these findings suggest that moderate inclusion of sunflower hulls, particularly at 40 g/kg, has the potential to enhance digestive efficiency, support gut health, and modulate microbial populations in broiler chickens. However, its efficacy in mitigating the performance drawbacks of reduced-crude protein diets warrants more cautious optimization depending on the specific dietary balanced amino acid profiles.

## 1 Introduction

In the modern poultry industry, optimizing feed efficiency and reducing dietary costs while maintaining bird performance and health remain paramount challenges. Crude protein (CP) is one of the most expensive components in broiler diets, and its optimization directly dictates the economic viability of poultry production. Reducing dietary CP levels not only minimizes formulation costs but also significantly mitigates environmental concerns by decreasing nitrogen excretion and improving litter quality (Kerr & Kidd, 1999). However, a drastic or poorly balanced reduction in CP often impairs growth performance, increases abdominal fat deposition, and compromises gastrointestinal development (Aletor et al., 2000).

Concurrently, Moderate inclusion of structural insoluble fiber is no longer viewed as a mere nutrient diluent, but rather as an essential functional component required to stimulate gastrointestinal development, enhance gizzard function, and improve nutrient digestibility (Mateos et al., 2012). Sunflower hulls (SFH) are an abundant agro-industrial by-product rich in insoluble fiber, particularly cellulose and lignin, making them an excellent candidate for improving the physical structure of the digestive tract. Also, it was shown that insoluble fiber concentration of 20–50 g/kg, can improve digestive efficiency without inducing negative side effects (Jimenez-Moreno et al., 2016; Kimiaeitalab et al., 2017).

It is hypothesized that the inclusion of optimal levels of insoluble fiber from SFH could counteract the negative impacts of low-CP diets by modifying intestinal mucosal architecture, modulating the cecal microflora, and enhancing overall nutrient utilization. However, comprehensive data regarding the interactive effects of low-CP diets and insoluble fiber sources on the gastrointestinal ecosystem and performance of broilers are limited. Therefore, this study was conducted to evaluate the interactive effects of varying levels of SFH and CP on growth performance, intestinal morphological traits (duodenum and jejunum), cecal microbial populations and apparent ileal nutrient digestibility in broiler chickens.

## 2 Materials and methods

### 2.1 Animal ethics

This study’s procedures were approved by the Animal Ethics Committee at the Agricultural Sciences and Natural Resources University of Khuzestan, Ahvaz, Iran (Approval no 40,325,604)

### 2.2 Sunflower hulls

Sunflower hulls were obtained from the by-products of the sunflower processing factory in Mashhad, Iran.

### 2.3 Diets

Table 1. Six diets were designed for broiler chickens to meet their nutrient requirements during different periods of growth: starter (0–10 d), grower (11–24 d), and finisher (25–42 d), following the suggestions provided for Ross 308 (Ross, 2022; Table 2). The treatments consisted of varying amounts of sunflower hulls (0, 20, and 40 g/kg) and protein levels: normal crude protein (NCP) and low crude protein (LCP) with a 10%-unit reduction in CP. The basal diet based on maize-soybean meal had 40 g/kg of sand as a chemically inert and non-nutritive and was replaced with 20 or 40 g/kg of sunflower hulls. All diets were in a mash form. To ensure that the SID amino acid levels were equivalent to those of the NCP diets, all LCP diets were supplemented with crystalline l-Lysine HCl, DL-Methionine, l-Threonine, l-Valine, l-Isoleucine, and l-Arginine (Table 2).

**Table 1:** Analyzed chemical composition of the sunflower hulls.

Parameter	g/kg

Dry matter	947.4
Crude protein	65.1
Ether extract	14.5
Ash	20.4
Crude fiber	490.9
NDF^1^	744.4
ADF^2^	528.7
NFC^3^	155.6
NFE^4^	409.1

1 NDF; Neutral Detergent Fiber,.

2 ADF; Acid Detergent fiber,.

3 Non-Fiber-Carbohydrate (NFC) = 100 - (CP + Ash + EE + NDF),.

4 Nitrogen free extract (NFE) = 100 - (CP + Ash + CF + EE).

**Table 2:** Composition, calculated analysis and analysed values (g/kg, as fed) of broiler starter, grower and finisher diets.

Item	**Starter (0–10)**									**Grower (11–24)**							**Finisher (25–42)**						
	Normal CP (23%)						Low CP (20.7%)			Normal CP (21.5%)			Low CP (19.35%)				Normal CP (19.5%)				Low CP (17.55%)		
	0 g/kg SFH			20 g/kg SFH		40 g/kg SFH	0 g/kg SFH	20 g/kg SFH	40 g/kg SFH	0 g/kg SFH	20 g/kg SFH	40 g/kg SFH		0 g/kg SFH	20 g/kg SFH	40 g/kg SFH	0 g/kg SFH	20 g/kg SFH	40 g/kg SFH	0 g/kg SFH		20 g/kg SFH	40 g/kg SFH
Ingredients, g/kg																							
Maize	442			442		442	526	526	526	482	482	482		544	544	544	524	524	524	568		568	568
Maize gluten meal (60%)	50			50		50	35	35	35	52	52	52		23	23	23	40	40	40	4		4	4
Soybean meal (42%)	378			378		378	310	310	310	323	323	323		299	299	299	297	297	297	285		285	285
Limestone	8.5			8.5		8.5	8.5	8.5	8.5	7.4	7.4	7.4		7.5	7.5	7.5	7.4	7.4	7.4	7.4		7.4	7.4
Dicalcium phosphate	15.0			15.0		15.0	15.8	15.8	15.8	13.0	13.0	13.0		13.0	13.0	13.0	10.4	10.4	10.4	10.5		10.5	10.5
Soybean oil	47.3			47.3		47.3	35.3	35.3	35.3	55.0	55.0	55.0		50.0	50.0	50.0	65.0	65.0	65.0	65.0		65.0	65.0
Sodium chloride	2.8			2.8		2.8	2.2	2.2	2.2	2.8	2.8	2.8		2.8	2.8	2.8	2.3	2.3	2.3	2.1		2.1	2.1
Sodium bicarbonate	2.3			2.3		2.3	3.3	3.3	3.3	2.3	2.3	2.3		2.3	2.3	2.3	2.0	2.0	2.0	2.3		2.3	2.3
Potassium carbonate	0.0			0.0		0.0	2.0	2.0	2.0	0.0	0.0	0.0		0.0	0.0	0.0	0.0	0.0	0.0	0.0		0.0	0.0
Mineral and vitamin premix^1^	5.0			5.0		5.0	5.0	5.0	5.0	5.0	5.0	5.0		5.0	5.0	5.0	5.0	5.0	5.0	5.0		5.0	5.0
DL-Methionine	3.2			3.2		3.2	3.8	3.8	3.8	2.7	2.7	2.7		3.6	3.6	3.6	2.6	2.6	2.6	3.3		3.3	3.3
L-lysine HCl	3.2			3.2		3.2	5.0	5.0	5.0	3.0	3.0	3.0		4.1	4.1	4.1	2.7	2.7	2.7	3.2		3.2	3.2
L-Threonine	1.4			1.4		1.4	2.3	2.3	2.3	1.0	1.0	1.0		1.7	1.7	1.7	0.8	0.8	0.8	1.4		1.4	1.4
L-Valine	0.5			0.5		0.5	1.6	1.6	1.6	0.0	0.0	0.0		1.0	1.0	1.0	0.0	0.0	0.0	0.8		0.8	0.8
L-Isoleucine	0.3			0.3		0.3	1.4	1.4	1.4	0.0	0.0	0.0		0.9	0.9	0.9	0.1	0.1	0.1	0.8		0.8	0.8
L-Arginine	0.6			0.6		0.6	2.4	2.4	2.4	0.4	0.4	0.4		1.5	1.5	1.5	0.3	0.3	0.3	0.9		0.9	0.9
Phytase	0.05			0.05		0.05	0.05	0.05	0.05	0.05	0.05	0.05		0.05	0.05	0.05	0.05	0.05	0.05	0.05		0.05	0.05
Washed sand^2^	40			20		0	40	20	0	40	20	0		40	20	0	40	20	0	40		20	0
SFH^4^	0			20		40	0	20	40	0	20	40		0	20	40	0	20	40	0		20	40
**Calculated analysis**																							
Metabolisable energy (Kcal/kg diet)		3004	3004		3004		2998	2998	2998	3104	3104	3104		3104	3104	3104	3204	3204	3204	3204		3204	3204
Crude protein		230	230		230		207	207	207	215.3	215.3	215.3		193.7	193.7	193.7	195.4	195.4	195.4	175.5		175.5	175.5
Ether extract		72	72		72		61.5	61.5	61.5	80.4	80.4	80.4		75.7	75.7	75.7	90.1	90.1	90.1	90.5		90.5	90.5
Calcium		9.6	9.6		9.6		9.6	9.6	9.6	8.6	8.6	8.6		8.5	8.5	8.5	7.9	7.9	7.9	7.9		7.9	7.9
Available phosphorus		4.7	4.7		4.7		4.8	4.8	4.8	4.3	4.3	4.3		4.3	4.3	4.3	3.9	3.9	3.9	3.9		3.9	3.9
Na		1.8	1.8		1.8		1.9	1.9	1.9	1.8	1.8	1.8		1.8	1.8	1.8	1.6	1.6	1.6	1.6		1.6	1.6
K		9.2	9.2		9.2		9.2	9.2	9.2	8.5	8.5	8.5		7.9	7.9	7.9	7.8	7.8	7.8	7.7		7.7	7.7
Cl		2.7	2.7		2.7		2.7	2.7	2.7	2.7	2.7	2.7		2.9	2.9	2.9	2.3	2.3	2.3	2.3		2.3	2.3
Arg (SID)^3^		13.5	13.5		13.5		13.4	13.4	13.4	12.3	12.3	12.3		12.1	12.1	12.1	11.1	11.1	11.1	10.9		10.9	10.9
Lys (SID)		12.5	12.5		12.5		12.4	12.4	12.4	11.4	11.4	11.4		11.4	11.4	11.4	10.3	10.3	10.3	10.3		10.3	10.3
Met (SID)		6.3	6.3		6.3		6.2	6.2	6.2	5.0	5.0	5.0		5.0	5.0	5.0	4.3	4.3	4.3	4.3		4.3	4.3
Met+Cys (SID)		9.3	9.3		9.3		9.3	9.3	9.3	8.6	8.6	8.6		8.7	8.7	8.7	8.0	8.0	8.0	7.9		7.9	7.9
Thr (SID)		8.5	8.5		8.5		8.5	8.5	8.5	7.7	7.7	7.7		7.6	7.6	7.6	6.9	6.9	6.9	6.8		6.8	6.8
Trp (SID)		2.3	2.3		2.3		2.3	2.3	2.3	2.1	2.1	2.1		1.9	1.9	1.9	1.9	1.9	1.9	1.8		1.8	1.8
Ile (SID)		8.6	8.6		8.6		8.6	8.6	8.6	7.8	7.8	7.8		7.6	7.6	7.6	7.1	7.1	7.1	7.0		7.0	7.0
Leu (SID)		18.4	18.4		18.4		18.3	18.3	18.3	12.7	12.7	12.7		12.7	12.7	12.7	11.3	11.3	11.3	11.3		11.3	11.3
Val (SID)		9.6	9.6		9.6		9.5	9.5	9.5	8.6	8.6	8.6		8.5	8.5	8.5	7.8	7.8	7.8	7.7		7.7	7.7
DCAB (mEq/kg)		2410	2410		2410		2410	2410	2410	2210	2210	2210		2010	2010	2010	2030	2030	2030	2000		2000	2000
Crude fiber		28.3	28.3		28.3		27.0	27.0	27.0	27.2	27.2	27.2		26.9	26.9	26.9	26.5	26.5	26.5	26.6		26.6	26.6
**Analysed values, g/kg**																							
Dry matter		902.8	931.3		933.0		901.1	930.2	932.7	929.9	931.9	921.7		928.9	929.1	928.5	907.8	921.4	918.4	908.3		911.1	914.8
Crude protein		231.2	233.5		234.3		202.1	203.0	208.0	217.0	219.0	220.0		189.0	190.0	195.0	196.8	198.8	199.8	170.5		171.5	176.5
Fat		71.5	70.2		73.5		62.0	63.0	62.5	81.0	81.3	80.0		74.9	74.8	76.0	89.0	89.9	90.2	90.0		90.6	91.0
Ash		60.6	98.8		53.3		60.0	60.2	61.0	82.2	85.7	85.0		90.8	91.5	77.7	84.6	76.2	74.3	80.9		91.0	88.9
Neutral detergent fiber		121.1	145.5		150.8		125.8	148.0	151.5	138.2	156.4	176.1		135.7	146.8	164.0	142.4	164.2	174.0	141.9		165.0	178.2
Acid detergent fiber		35.2	37.1		42.0		26.8	38.4	45.5	55.2	63.8	67.0		54.4	39.0	53.2	36.9	61.5	56.6	39.2		56.3	67.1

1 Premix provided per kilogram of diet: Fe, 60 mg; Mn, 100 mg; Zn, 60 mg; Cu, 10 mg; I, 1 mg; Co, 0.2 mg; Se, 0.15 mg; retinyl acetate, 1.55 mg; cholecalciferol, 0.025 mg; α-tocopherol acetate, 20 mg; menadione, 1.3 mg; thiamine, 2.2 mg; riboflavin, 10 mg; calcium pantothenate, 10 mg; choline chloride, 400 mg; nicotinamide, 50 mg; pyridoxine HCl, 4 mg; biotin, 0.04 mg; folic acid, 1 mg; vitamin B12 (cobalamin), 1.013 mg.

2 Sand was replaced by 20 and 40 g/kg sunflower hulls in experimental diets.

3 SID: standardized ileal digestible.

4 SFH: sunflower hulls.

### 2.4 Birds and housing

A total of 360 one-day-old Ross 308 broiler chicks, consisting of both males and females, were acquired from a commercial hatchery. Upon arrival, the mean body weight was 40.0 ± 1.5 *g*Chicks were immediately assigned to 36 floor pens in a completely randomized design with 3 × 2 factorial arrangement, ensuring a uniform initial body weight across all pens. Each treatment had six replicates with 10 birds in each. Throughout the 42-day experimental period, birds were managed according to the Ross broiler management handbook (Aviagen, 2018), with ad libitum access to the experimental diets and fresh water. Each pen had one drinker and one feeder suspended inside. Fresh wood shavings were used as bedding material. The broiler chickens were initially placed at a temperature of 33 °C, which was then gradually lowered to 22 ^°^C by day 42. The vaccination program for broiler chickens during the rearing period was implemented as follows: At 7 days of age, birds received the Newcastle disease (B1) vaccine via eye drops, and a vaccine against avian influenza and infectious bronchitis was administered by subcutaneous injection in the neck. At 21 days of age, a Newcastle disease vaccine was provided through the drinking water.

### 2.5 Performance data

The birds' body weight gain (BWG) was calculated for each pen on days 1, 10, 24, and 42. Feed intake (FI) per pen was measured on days 10, 24, and 42 by subtracting the remaining feed after each feeding period from the total feed given, and the mean FCR (g/g) of the flock was determined. Mortality was documented on a daily basis for the duration of the experiment.

### 2.6 Determination of apparent ileal digestibility

Chromium oxide (3.0 g/kg; Reachim, USSR) was included to the diets of grower period (18–25 days old) as an undigestible marker to measure ileal nutrient digestibility. On the 25th day, two birds in each replicate were euthanized with cervical dislocation and the contents of the ileum, from Meckel's diverticulum to the ileocaecal junction, were collected by carefully squeezing them into sealed bags. The specimens were stored at a frozen temperature of −20 °C until they were analyzed.

### 2.7 Antibody response

To examine the primary and secondary humoral immune response, two birds per replicate were injected with 0.25 mL of a 10% sheep red blood cell (SRBC) suspension in phosphate-buffered saline on days 14 and 35 (Rostami et al., 2015). Blood samples (2 ml) were taken from the broiler chickens' brachial vein at 7 days following each injection. The serum was isolated through centrifugation (3000 × g, 10 min) and preserved at −20 °C for future analysis. The overall level of anti-SRBC antibodies was measured using a hemagglutination technique with a round-bottomed 96-well microplate as described in Wegmann and Smithies' (1966) study. Each sample's Anti-SRBC titer was calculated as log^2^ of the reciprocal of the highest dilution of serum that showed detectable agglutination (Akhavan-Salamat & Ghasemi, 2019).

### 2.8 Gut morphology and cecal microbial populations

At 42 days of age, two birds were chosen from each replicate, based on the average weight of the replicate and were then euthanized by cervical dislocation. Subsequently, a 2 cm segment was excised from the center of the duodenum, jejunum and ileum for histological analysis. The samples were rinsed with distilled water and preserved in a 10% formalin solution. The samples fixed in formalin were prepared for embedding in paraffin. 5 µm slices were acquired with a rotary microtome (Model: JUNG RM 2055, Leica Microsystems GmbH, Wetzlar, Germany). Sections were stained with hematoxylin-eosin to be examined under light microscopy. The villus height and crypt depth were used to determine the V:C ratio, using the methodology described by Baurhoo et al. (2009). Muscle thickness was measured on the same H&E-stained sections using a light microscope equipped with a digital image analysis system (ImageJ, NIH, Bethesda, MD, USA). Measurements were taken from the serosa to the inner border of the submucosa at three different points per section, and the mean value was calculated, as described by Duque-Ramírez et al. (2023).

On the 42th day, two birds from each replicate were selected and their caecal digesta was immediately placed on ice until being transported to the lab for microbial population counting (Pourazadi et al., 2020). The contents from the caecum (1 g) of each bird were transferred into 9 ml of sterile saline solution and diluted from 10 ^−2^ to 10 ^−10^. *Lactobacilli* were counted on MRS agar and *Escherichia coli*and *Coliforms* were enumerated on Mac Conkey (MC) agar following incubation at 37 °C for 48 h in an anaerobic chamber and for 24 h in an aerobic chamber, respectively (Guban et al., 2006). Each sample was plated twice.

### 2.9 Digesta transit time and characteristics of broiler litter

The transit time of digesta was established when the chicks were 21 days old. All diets contained 3 g/kg of Chromium oxide (Cr_2_O_3_) as an indigestible marker. Birds were given limited access to feed for 30 min prior to being given feed with the marker. The duration between giving birds feed with added Cr_2_O_3_ and the first appearance of green color in faeces determined the transit time (min) (Dos Santos et al., 2017). Litter samples were collected from five different locations within each pen on day 42, pooled thoroughly, and subsequently analyzed for moisture, nitrogen, and dry matter contents (Ball et al., 2024).

### 2.10 Chemical analysis

Prior to chemical analysis, samples of feed and ileal digesta were pre-dried in a oven at 60 °C for 72 h and then ground to pass through a 0.5 mm screen. Similarly, for litter characterization, pooled samples were weighed and dried at 105 °C for 24 h to determine moisture and dry matter content, with the weight difference representing the moisture percentage. Samples of sunflower hulls (SFH), experimental diets, and ileal digesta were analyzed for dry matter (DM), crude protein (CP; N × 6.25), ether extract (EE), and ash. Additionally, SFH and diets were analyzed for crude fiber (CF), while neutral detergent fiber (NDF) and acid detergent fiber (ADF) were determined for SFH only. All nutrient analyses were conducted according to the official methods of AOAC (2000) as follows: DM (Method 930.15), CP (Kjeldahl technique; Method 990.03), EE (Soxhlet method after acid hydrolysis; Method 954.02), CF (Method 978.10), and Ash (Method 942.05). The NDF and ADF contents were measured according to Van Soest et al. (1991) and reported on an ash-free basis. The chromium oxide concentration in the diets and ileal digesta was determined using an atomic absorption spectrophotometer (ContrAA 300, Analytik Jena, Germany) following the procedure described by Saha & Gilbreath (1991). Litter nitrogen was determined using the Kjeldahl device according to AOAC (2000) standards.

### 2.11 Calculations

The AID of nutrients was calculated using the following formula: (Hafeez et al., 2016):

AID (%) = 100 − ((concentration of marker in feed/concentration of marker in digesta) × (concentration of nutrient in digesta/ concentration of nutrient in feed) × 100).

### 2.12 Statistical analysis

The replicated pen served as the experimental unit. Two-way ANOVA was utilized to analyze the data in order to investigate the main effects (SFH and CP levels) and their interaction, using the GLM procedure of SAS (2015). The significance level was established at P < 0.05, and significant differences between means were distinguished using Duncan's multiple range tests (Duncan, 1955). Assumptions of normality of the residuals (Shapiro-Wilk test) and homogeneity of variances were verified and met before performing the ANOVA.

The statistical model was as follows:

$$y_{ijk}=\mu +O_{i}+P_{j}+{\left(OP\right)}_{ij}+e_{ijk}$$

Where:

*y*_ijk_: observed response; μ= the population mean.

O_i_: sunflower hulls levels effect (i = 1, 2 and 3), P_j_: crude protein levels effect (j = 1 and 2), (OP)_ij_: Interaction of sunflower hulls levels × crude protein levels. e _ijk_: random error.

## 3 Results

### 3.1 Growth performance

The mortality rate of about 3% observed during the study was not dependent on any specific dietary treatment.

**Fig. 1:**
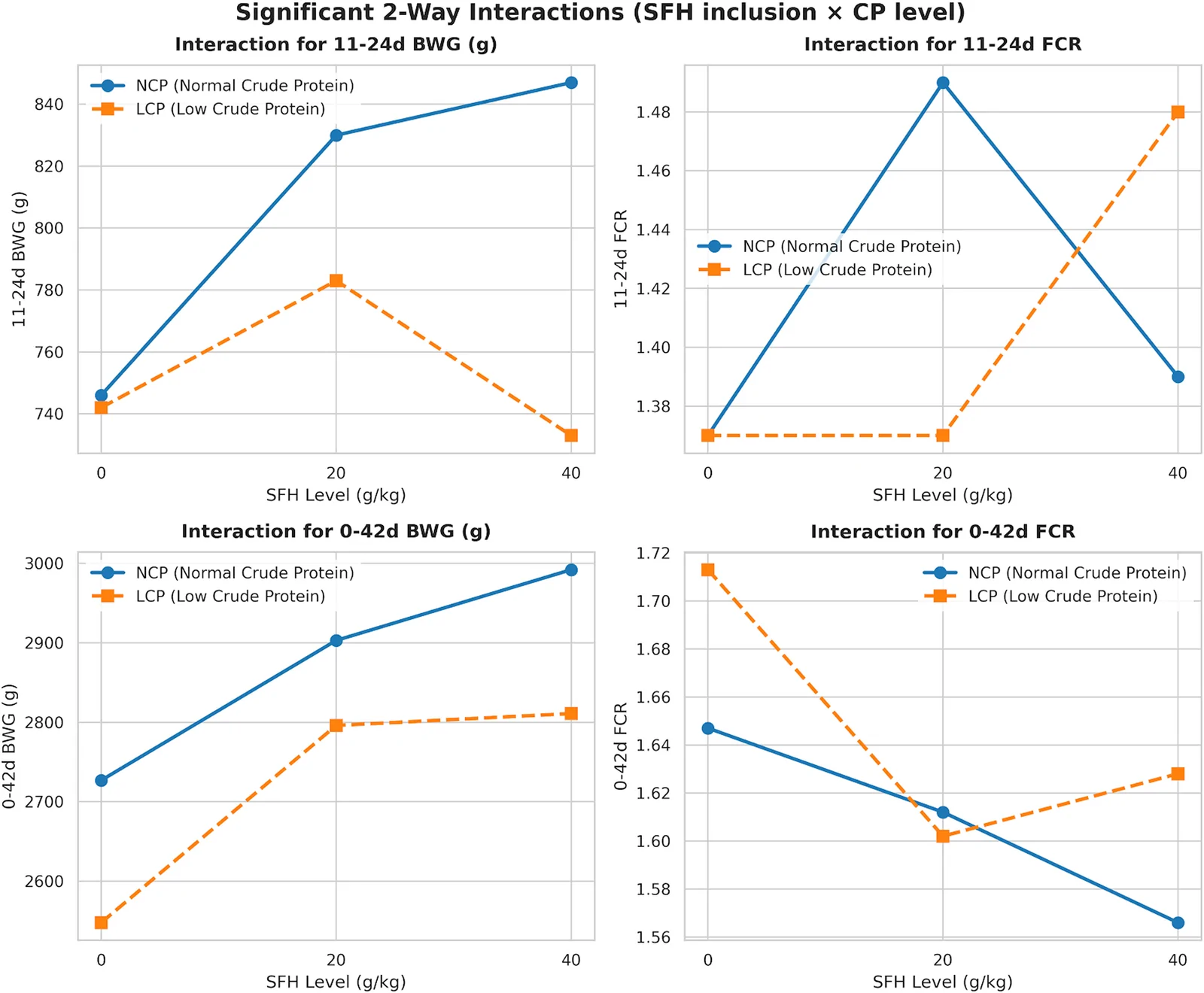
Effect of significant 2-way interactions (dietary sunflower hulls (SFH) and protein density) on body weight gain (BWG) and feed conversion ratio (FCR) of broiler chickens.

During the initial phase (0–10 d), the introduction of 20 g/kg SFH provided a beneficial structural stimulus, leading to superior body weight gain (BWG) compared to fiber-free controls (Table 3, Fig. 1). Throughout the entire experimental period (0–42 d), maintaining normal protein levels (NCP) consistently supported higher feed intake and growth rates. The reduction of dietary protein (LCP) typically compromises performance, the inclusion of SFH mitigated this decline. Broilers fed diets containing 40 g/kg SFH alongside normal protein achieved the most efficient feed conversion ratio (FCR), whereas the absence of fiber in low-protein diets resulted in the poorest efficiency.

**Table 3:** Interactive effects of dietary sunflower hulls (SFH) and protein density on feed intake (FI, g/bird), body weight gain (BWG, g/bird), feed conversion ratio (FCR, g/g) of broiler chickens during 0–42 days of age^a^.

Days post-hatch													
		0–10 d			11–24 d			25–42 d				0–42 d	
Level of SFH (g/kg)	CP level	BWG	FI	FCR	BWG	FI	FCR	BWG	FI	FCR	BWG	FI	FCR
0	NCP	216	235	1.08 ^bc^	746 ^c^	1029^c^	1.37 ^c^	1765^d^	3228	1.82 ^b^	2727 ^d^	4492	1.64 ^b^
	LCP	208	222	1.06 ^c^	742^c^	1020 ^c^	1.37 ^c^	1597^e^	3121	1.95 a	2548 ^e^	4365	1.71 a
20	NCP	231	242	1.04 ^c^	830^a^	1212^a^	1.49 a	1842^b^	3224	1.75 ^c^	2903 ^b^	4679	1.61 ^cd^
	LCP	211	240	1.13 a^b^	783 ^b^	1073 ^b^	1.37 ^c^	1802 ^c^	3164	1.75 ^c^	2796^c^	4478	1.60 ^d^
40	NCP	223	245	1.09 ^bc^	847 a	1178^a^	1.39 ^bc^	1921^a^	3261	1.70 ^d^	2992^a^	4685	1.56 ^e^
	LCP	208	241	1.15 a	733 ^c^	1082^b^	1.48 a^b^	1869^b^	3252	1.74 ^cd^	2811^c^	4576	1.62 ^bc^
SEM		2.5	4.6	0.020	11.3	11.7	0.024	10.9	19.4	0.015	12.5	22.4	0.010
**Main effects**													
Levels of SFH													
0		212 ^b^	228 ^b^	1.07	744^b^	1025^b^	1.37^b^	1681^c^	3174^b^	1.88^a^	2638^c^	4428^c^	1.67^a^
20		221 a	241 a	1.09	806^a^	1143^a^	1.41^b^	1822^b^	3194 ^b^	1.75^b^	2850^b^	4579^b^	1.60^b^
40		215 a^b^	243 a	1.13	786^a^	1130^a^	1.43^a^	1895^a^	3256^a^	1.71^c^	2897^a^	4630^a^	1.59^b^
CP level													
	NCP	223 a	240	1.07^b^	808^a^	1140^a^	1.41	1843^a^	3338^a^	1.76^a^	2874^a^	4619^a^	1.60^b^
	LCP	209 ^b^	235	1.12^a^	750^b^	1059^b^	1.41	1756^b^	3179^b^	1.81^b^	2715^b^	4473^b^	1.64^a^
**Probabilities, P ≤**													
SFH inclusion		0.007	0.007	0.058	>0.0001	>0.0001	0.039	>0.0001	0.0005	>0.0001	>0.0001	>0.0001	>0.0001
CP level		>0.0001	0.145	0.008	>0.0001	>0.0001	0.784	>0.0001	0.0008	>0.0001	>0.0001	>0.0001	>0.0001
SFH inclusion × CP level		0.066	0.479	0.023	>0.0001	>0.0001	0.002	>0.0001	0.059	0.0025	0.004	0.1132	0.0012

SFH, sunflower hulls; NCP, Normal crude protein; LCP, Low crude protein; SEM, Standard error of means.

a Data are mean values of 6 replicate cages per treatment (10 birds per replicate cage). Means in a column not sharing a common letter (a-d) are significantly different (P < 0.05).

### 3.2 Nutrient digestibility

**Fig. 2:**
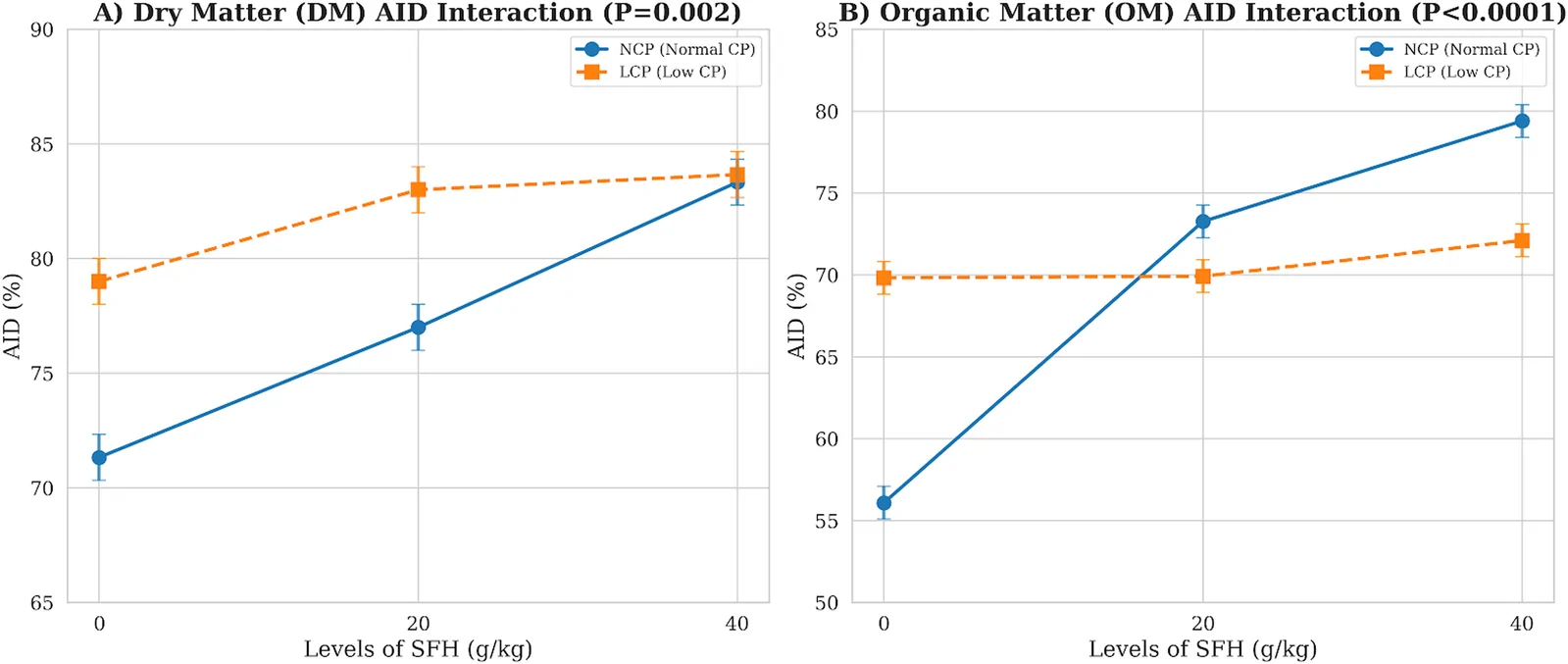
Effect of significant 2-way interactions (dietary sunflower hulls (SFH) and protein density) on organic matter and dry matter digestibility in broiler chickens.

High levels of SFH (40 g/kg) led to the highest apparent ileal digestibility (AID) of nitrogen and organic matter (Table 4). An important interaction was noted for dry matter (DM) digestibility: while fiber-free low-protein diets showed poor DM digestibility, the addition of 40 g/kg SFH to these diets significantly improved digestibility levels (Fig. 2). Furthermore, ether extract (EE) digestibility was improved in reduced-protein diets and improved further with fiber inclusion.

**Table 4:** Interactive effects of dietary sunflower hulls (SFH) and protein density on the apparent ileal digestibility (AID) of nutrients in broiler chickens at 25 days of age^a^.

		**AID (%)**			
Levels of SFH (g/kg)	CP	DM	N	EE	OM
0	NCP	71.3 ^c^	61.1	64.8	56.0 ^c^
	LCP	79.0 ^b^	59.9	68.1	69.8 ^b^
20	NCP	77.0 ^b^	69.8	74.8	73.2 ^b^
	LCP	83.0 a	68.9	75.4	69.9 ^b^
40	NCP	83.3 a	73.5	72.0	79.4 a
	LCP	83.6 a	73.3	79.6	72.1 ^b^
SEM		0.008	0.008	0.015	0.011
**Main effects**					
Levels of SFH					
0		75.1 ^c^	60.5 ^c^	66.4 ^b^	62.9 ^c^
20		80.0 ^b^	69.3 ^b^	75.1 a	71.5 ^b^
40		83.5 a	73.4 a	75.8 a	75.7 a
CP levels					
	NCP	77.2 ^b^	68.1	70.6 ^b^	69.5
	LCP	81.8 a	67.3	74.3 a	70.6
**Probabilities, P ≤**					
SFH inclusion		0.0001	0.0001	0.0008	0.0001
CP level		0.0001	0.556	0.037	0.429
SFH inclusion × CP level		0.002	0.946	0.243	0.0001

SFH, sunflower hulls; NCP, Normal crude protein; LCP, Low crude protein; SEM, Standard error of means; DM, Dry matter; EE, Ether extract; OM, Organic matter.

a Data are mean values of 6 replicate per treatment (2 birds per replicate). Means in a column not sharing a common letter (a-c) are significantly different (P < 0.05).

### 3.3 Immunity response

Neither SFH levels nor CP level and SFH and CP levels interaction showed significant effect (P < 0.05) on primary and secondary immune responses (Table 5).

**Table 5:** Interactive effects of dietary sunflower hulls (SFH) and protein density on the anti-sheep red blood cell (SRBC) antibody response of broilers.

Levels of SFH	CP	Primary	Secondary
0	NCP	4.50	5.33
	LCP	4.16	6.00
20	NCP	4.50	5.30
	LCP	4.83	6.16
40	NCP	4.33	5.66
	LCP	5.16	5.83
SEM		0.400	0.392
**Main effects**			
Levels of SFH			
0		4.75	5.66
20		4.66	5.70
40		4.33	5.75
CP levels			
	NCP	4.44	5.44
	LCP	4.72	6.00
**Probabilities, P ≤**			
SFH inclusion		0.55	0.97
CP level		0.40	0.09
SFH inclusion × CP level		0.35	0.67

SFH, sunflower hulls NCP, Normal crude protein; LCP, Low crude protein; SEM, Standard error of means.

^a^ Data are mean values of 6 replicate cages per treatment (2 birds per replicate cage).

Means in a column not sharing a common letter (a-d) are significantly different (P < 0.05).

### 3.4 Intestinal morphology

Increasing SFH levels progressively enhanced duodenal villus height and muscle layer thickness, effectively expanding the absorptive surface area (Table 6).

**Table 6:** Interactive effects of dietary sunflower hulls (SFH) and protein density on the intestinal morphology of broiler chickens on d 42.

		Duodenum						Jejunum						Ileum						
		Villus Height (μm)	Villus Width (μm)	Villus Area c (mm2)	Muscle layer Thickness(μm)	Crypt Depth (μm)	V:C b	Villus Height (μm)	Villus Width (μm)	Villus Area c (mm2)	Muscle layer Thickness(μm)	Crypt Depth (μm)	V:C b	Villus Height (μm)	Villus Width (μm)	Villus Area c (mm2)	Muscle layer Thickness(μm)		Crypt Depth (μm)	V:C b
Levels of SFH	CP																			
0	NCP	1361	115.5 ^e^	0.49 ^d^	156.3	141	9.63	1045	126.1	0.41	208.1	134	7.77	773	152.7	0.37	190.9		130	5.98
	LCP	1351	123.6 ^d^	0.52 ^d^	160.2	144	9.34	989	120.6	0.37	189.3	148	6.67	718	151.5	0.34	180.6		125	5.76
20	NCP	1407	131.0 c	0.57 c	161.6	152	9.22	1098	129.3	0.44	215.9	133	8.24	881	152.4	0.42		214.0	131	6.58
	LCP	1395	134.1 c	0.58 c	164.2	152	9.20	1030	121.7	0.39	212.5	138	7.42	900	154.1	0.43		186.8	134	6.68
40	NCP	1476	154.3 a	0.71 a	169.6	157	9.38	1125	131.0	0.46	234.6	134	8.41	968	155.4	0.47		181.7	135	7.17
	LCP	1449	140.7 b	0.64 b	166.8	146	9.88	1046	125.9	0.41	218.4	136	7.69	906	157.2	0.44		202.1	141	6.42
SEM		14.30	1.73	0.01	1.62	3.70	0.22	5.60	1.57	0.006	6.76	2.50	0.49	22.10	3.03	0.01		10.70	5.20	0.24
**Main effects**																				
Levels of SFH																				
0		1356^c^	119.5 c	0.50 c	158.3 c	143^b^	9.48	1017 c	123.4 b	0.39 c	198.7 b	141	7.22 b	746^b^	152.1	0.35 c		185.7	127	5.87 b
20		1401 b	132.5 b	0.58 b	162.9 b	152^a^	9.21	1064^b^	125.5 a^,^^b^	0.42 b	214.2 a	136	7.83 a	891^a^	153.2	0.42 b		200.4	133	6.63 a
40		1463^a^	147.5 a	0.67 c	168.2 a	152 a	9.63	1086^a^	128.4 a	0.43 a	226.5 a	135	8.05 a	937^a^	156.3	0.46 a		191.9	138	6.80 a
CP levels																				
NCP		1415	133.6	0.59	162.5	150	9.41	1089^a^	128.8 a	0.44 a	219.5 a	133 b	8.14 a	874	153.5	0.42		195.5	132	6.58
LCP		1398	132.8	0.58	163.7	147	9.47	1022^b^	122.7 b	0.39 b	206.7 b	141 a	7.26 b	841	154.3	0.40		189.8	133	6.29
**Probabilities, P ≤**																				
SFH inclusion		<0.0001	<.0001	<.0001	<.0001	0.036	0.196	<.0001	0.016	<.0001	0.002	0.061	0.0001	<.0001	0.376	<.0001		0.406	0.177	0.002
CP level		0.176	0.582	0.175	0.366	0.391	0.732	<.0001	0.0002	<.0001	0.032	0.003	<.0001	0.087	0.746	0.184		0.522	0.758	0.163
SFH inclusion × CP level		0.809	<.0001	0.000	0.126	0.154	0.234	0.165	0.697	0.529	0.487	0.095	0.463	0.151	0.860	0.177		0.106	0.583	0.230

SFH, sunflower hulls; NCP, Normal crude protein; LCP, Low crude protein; SEM, Standard error of means.

a Data are mean values of 6 replicate cages per treatment (2 birds per replicate cage). Means in a column not sharing a common letter (a-d) are significantly different (P < 0.05).

b V:C = villus height to crypt depth ratio.

c Villus area was calculated by multiplying villus height with width at half height.

The interaction between fiber and protein was particularly evident in the duodenal and jejunal structures. The combination of 40 g/kg SFH and normal protein supported the greatest villus width and area. Conversely, protein deficiency (LCP) tended to shorten villi and deepen crypts. The use of 40 g/kg SFH helped counteract these negative shifts, maintaining a more robust intestinal barrier.

### 3.5 Caecal microbial populations

Sunflower hulls inclusion levels significantly (P < 0.05) affected the caecal microbiota in broilers. Caecal microbial population of *Lactobacilli* spp. increased by increasing SFH level (Table 7). Crude protein level had significant effect on *Coliform* population. As dietary crude protein decreased, the number of *Coliform* bacteria increased significantly (P < 0.01), while there was no impact on *E.coli*levels with varied diets (P > 0.05).

**Table 7:** Interactive effects of dietary sunflower hulls (SFH) and protein density on the caecal microbial population (log cfu g^−1^)^1^ of broiler chickens on d 42.

Levels of SFH	CP	*Lactobacilli* spp.	*Coliforms*	*E. coli*
0	NCP	8.26	6.81	6.18
	LCP	8.29	6.89	6.08
20	NCP	9.08	6.68	6.25
	LCP	9.07	7.07	6.38
40	NCP	9.34	6.70	6.14
	LCP	9.22	6.87	6.37
SEM		0.046	0.085	0.176
**Main effects**				
Levels of SFH				
0		8.28 ^c^	6.85	6.13
20		9.08 ^b^	6.87	6.31
40		9.28 a	6.78	6.26
CP levels				
	NCP	8.89	6.73 ^b^	6.19
	LCP	8.86	6.94 a	6.28
**Probabilities, P ≤**				
SFH inclusion		0.0001	0.57	0.57
CP level		0.59	0.005	0.53
SFH inclusion × CP level		0.45	0.20	0.63

SFH, sunflower hulls; NCP, Normal crude protein; LCP, Low crude protein; SEM, Standard error of means.

a Data are mean values of 6 replicate cages per treatment (2 birds per replicate cage). Means in a column not sharing a common letter (a-c) are significantly different (P < 0.05).

### 3.6 Characteristics of broiler litter and digesta transit time

The impact of dietary treatments on litter moisture did not yield statistically significant results (P > 0.05; Table 8). However, litter nitrogen was lower (P < 0.01) at 40 g/kg SFH than control and 20 g/kg SFH. Moreover, reducing dietary CP led to a notable (P < 0.05) decrease in litter N content. Interaction SFH inclusion × CP level was significant (P > 0.001) for digesta transit time. There was no impact of protein level on the transit time of digesta in birds fed 0 and 40 g/kg SFH, on the other hand, broilers that were given 20 g/kg experienced longer digesta transit times with LCP than with NCP.

**Table 8:** Interactive effects of dietary sunflower hulls (SFH) and protein density on litter moisture and nitrogen and digesta transit time of broiler chickens on d 42.

**Levels of SFH**	**CP**	**Litter moisture %**	**Litter nitrogen(% DM)**	**Digesta transit time (min)**
0	NCP	61.91	3.19	166.66 ^b^
	LCP	64.10	3.00	166.83 ^b^
20	NCP	64.71	3.08	177.50 ^b^
	LCP	61.05	2.77	203.33 ^a^
40	NCP	63.65	2.71	210.00 ^a^
	LCP	63.18	2.43	200.00 ^a^
SEM		1.490	0.105	6.379
**Main effects**				
Levels of SFH				
0		63.00	3.09 ^a^	166.26 ^c^
20		62.88	2.92 ^a^	190.41 ^b^
40		63.42	2.57 ^b^	205.00 ^a^
CP levels				
	NCP	63.41	2.99 ^a^	184.72
	LCP	62.74	2.73 ^b^	189.72
**Probabilities, P ≤**				
SFH inclusion		0.93	0.0003	0.0001
CP level		0.60	0.007	0.344
SFH inclusion × CP level		0.17	0.832	0.023

SFH, sunflower hulls; NCP, Normal crude protein; LCP, Low crude protein; SEM, Standard error of means.

Means in a column not sharing a common letter (a-c) are significantly different (P < 0.05).

## 4 Discussion

The significant interaction observed between SFH inclusion and CP levels on BWG and FCR over the 42-day period highlights that the utilization of low-CP diets is fundamentally dependent on structural material in the diet. Rather than a simple dilution effect, the addition of 40 g/kg SFH to NCP diets optimized nutrient utilization, while its absence in LCP diets resulted in the poorest FCR. Statistically, reducing dietary CP is known to compromise broiler performance due to a potential imbalance in non-essential amino acids or altered energy-to-protein dynamics (Hilliar et al., 2020). However, our data critically demonstrates that the inclusion of SFH mitigates these negative effects. Physiologically, this can be explained by the physical stimulation of the gizzard. The accumulation of insoluble fiber components in the gizzard triggers muscular hypertrophy and prolongs digesta retention (Mateos et al., 2012). This development triggers a more robust secretion of hydrochloric acid and pepsin, lowering gizzard pH and improving pepsin activity lowering gizzard pH and maximizing proteolytic activity (Jiménez-Moreno et al., 2009).

Crucially, these gizzard-driven physiological adaptations directly underpin the observed improvements in the apparent ileal digestibility (AID) of N, OM, and EE. The enhanced mechanical grinding and longer retention time in a well-developed gizzard ensure that the digesta entering the small intestine is more thoroughly broken down and exposed to endogenous enzymes. Furthermore, the lower gizzard pH not only optimizes gastric protein hydrolysis but also potentially triggers downstream pancreatic enzyme secretions and slows down intestinal transit rate, thereby maximizing the time available for nutrient absorption in the ileum. Therefore, the improved nutrient digestibility with the inclusion of 40 g/kg SFH is a direct consequence of this enhanced gastrointestinal functionality.

The structural modifications observed in intestinal architecture, specifically the linear increase in villus height and total mucosal absorptive area in the duodenum and jejunum, provide the anatomical justification for the enhanced nutrient digestibility coefficients. Our histomorphological data strongly refutes this abrasion hypothesis, as crypt depth remained unaltered across the fiber-supplemented groups. Rather than causing pathological tissue damage, the particles of SFH exert a mild, beneficial mechanical shearing against the intestinal wall, which stimulates cell proliferation along the crypt-villus axis and accelerates mucosal development (Rahmatnejad & Saki, 2016). Conversely, the reduction in villus height observed in LCP diets without SFH indicates an anatomical down-regulation driven by a scarcity of essential amino acids required for enterocyte protein synthesis (Laudadio et al., 2012). The inclusion of SFH successfully rescued this structural deficit, proving that mechanical stimulation can partially compensate for marginal nutritional deficiencies by maximizing the available absorptive surface. Moreover, recent evidence indicates that dietary fiber-based interventions can improve ileal digestibility, gut health, and intestinal morphology in broilers, supporting the beneficial role of appropriately formulated fiber sources in poultry nutrition (Islam et al., 2024; Ahmad et al., 2024; Sultan et al., 2024).

The significant proliferation of cecal *Lactobacillus spp*. in response to higher SFH levels highlights an interesting microbial dynamic. Although SFH consists primarily of lignified, non-fermentable carbohydrate fractions, its ability to increase digesta retention time allows the cecal commensal microflora sufficient time to partially ferment the structural matrix. This low-rate fermentation produces volatile fatty acids (VFAs), which lower the luminal pH and establish a competitive exclusion environment that suppresses pathogenic enterobacteria. In LCP diets, less undigested protein enters the hindgut, fundamentally depriving uric acid-producing and proteolytic bacteria of their substrate (Hilliar et al., 2020). When coupled with SFH, the enhanced upstream ileal N digestibility further restricts the nitrogenous substrate reaching the ceca. In addition, soybean hull supplementation can support the broader concept that structural fibrous ingredients can positively influence gastrointestinal function and microbial balance (Shuaib et al., 2022).

Regarding the interaction between SFH inclusion and CP levels on digesta passage rate, our results demonstrated that the dietary inclusion of sunflower hulls prolonged the transit time specifically at the 20 g/kg inclusion level, rather than exerting a general linear effect across all treatments. This specific delay in digesta transit time under the 20 g/kg SFH diet might provide a longer window for enzyme-substrate interactions in the upper gastrointestinal tract, potentially correlating with the minor qualitative shifts in fermentation and nutrient utilization observed in this study. However, the lack of a similar significant effect at the higher inclusion level (40 g/kg) suggests that the physiological response of passage rate to insoluble fiber matrix in broilers may not follow a strictly linear pattern, requiring caution in generalized conclusions (Hetland et al., 2004).

## 5 Conclusions

Dietary inclusion of sunflower hulls at 20 or 40 g/kg improved nutrient digestibility, intestinal morphology, and cecal microbiota in broilers. The most effective treatment was 40 g/kg SFH combined with normal crude protein, which maximized growth performance and reduced litter nitrogen excretion. While these results demonstrate the functional benefits of SFH, future research should focus on evaluating economic feasibility and refining dietary recommendations under diverse commercial environments to optimize performance for modern broiler strains.

## Ethical Statement

The authors confirm that the ethical policies of the journal, as noted on the journal's author guidelines page, have been adhered to and the appropriate ethical review committee approval has been received. The authors confirm that they have followed EU standards for the protection of animals used for scientific purposes.

## Ethics declaration

This study was conducted in accordance with the following guidelines for animal welfare and/or reporting: This study’s procedures were approved by the Animal Ethics Committee at the Agricultural Sciences and Natural Resources University of Khuzestan, Ahvaz, Iran (Approval no 40325604). This study was approved by the Agricultural Sciences and Natural Resources University of Khuzestan (Approval No. 40325604).

## CRediT authorship contribution statement

**N. Beigzadeh:** Writing – original draft, Software, Investigation. **S. Salari:** Writing – review & editing, Methodology. **F. Zaefarian:** Writing – review & editing, Methodology. **Sh. Hosseinifar:** Methodology.

## Declaration of competing interest

The authors declare no conflict of interest.
